# In silico analysis identifies novel restriction enzyme combinations that expand reduced representation bisulfite sequencing CpG coverage

**DOI:** 10.1186/1756-0500-7-534

**Published:** 2014-08-15

**Authors:** Daniel B Martinez-Arguelles, Sunghoon Lee, Vassilios Papadopoulos

**Affiliations:** The Research Institute of the McGill University Health Centre, Montreal General Hospital, 1650 Cedar Avenue, Room C10-143, Montréal, Québec H3G 1A4 Canada; Department of Medicine, McGill University, Montreal, QC Canada; Departments of Biochemistry, McGill University, Montreal, QC Canada; Departments of Pharmacology & Therapeutics, McGill University, Montreal, QC Canada

**Keywords:** RRBS, DNA methylation, Epigenetics, Restriction enzyme, Next-generation sequencing

## Abstract

**Background:**

Epigenetics is the study of gene expression changes that are not caused by changes in the deoxyribonucleic acid (DNA) sequence. DNA methylation is an epigenetic mark occurring in C–phosphate–G sites (CpGs) that leads to local or regional gene expression changes. Reduced-representation bisulfite sequencing (RRBS) is a technique that is used to ascertain the DNA methylation of millions of CpGs at single-nucleotide resolution. The genomic coverage of RRBS is given by the restriction enzyme combination used during the library preparation and the throughput capacity of the next-generation sequencer, which is used to read the generated libraries. The four-nucleotide cutters, *MspI* and *TaqαI*, are restriction enzymes commonly used in RRBS that, when combined, achieve ~12% genomic coverage. The increase in throughput of next-generation sequencers allows for novel combinations of restriction enzymes that provide higher CpG coverage.

**Results:**

We performed a near-neighbor analysis of the four nucleotide sequences most frequently found within 50 nt of all genomic CpGs. This resulted in the identification of seven methylation-insensitive restriction enzymes (*AluI*, *BfaI*, *HaeIII*, *HpyCH4V*, *MluCI*, *MseI*, and *MspI*) that shared similar restriction conditions suitable for RRBS library preparation. We report that the use of two or three enzyme combinations increases the theoretical epigenome coverage to almost half of the human genome.

**Conclusions:**

We provide the enzyme combinations that are more likely to increase the CpG coverage in human, rat, and mouse genomes.

**Electronic supplementary material:**

The online version of this article (doi:10.1186/1756-0500-7-534) contains supplementary material, which is available to authorized users.

## Background

Epigenetics is the study of gene expression changes that are not caused by changes in the deoxyribonucleic acid (DNA) sequence. Methylation of cytosine at CG dinucleotides is an epigenetic mark that is shown to modulate local and regional gene expression [1]. Multiple techniques have been developed to quantify DNA methylation, which center around the treatment of DNA with bisulfite, the use of restriction enzymes sensitive to DNA methylation, or the use of methylation-binding proteins [2]. The reduced-representation bisulfite sequence (RRBS) is a robust technique that provides DNA methylation levels at a nucleotide resolution of millions of CpGs with little DNA input [3]. RRBS is becoming increasingly popular because it provides a higher resolution and greater genomic coverage than array-based technologies, and it is cheaper than whole-genome bisulfite sequencing. The CpG coverage of RRBS has been improved by the increase in sequencing throughput and the depth of sequencing of next-generation sequencers (NGS).

RRBS was originally described as using a DNA methylation-insensitive restriction enzyme with a consensus sequence that is often found in C–phosphate–G (CpG)-rich regions to digest genomic DNA. The fragments that are generated are selected for size and contain a “reduced representation” of the starting genomic DNA. The size-selected fragments are ligated to chemically modified sequencing adapters, treated with bisulfite, and are amplified via polymerase chain reaction (PCR). The resulting RRBS library continues to a standard next-generation sequencing pipeline. The sequencing reads are aligned against a reference genome, while DNA methylation levels are ascertained by counting the frequency of CGs (methylated) and TGs (demethylated) at the various CpG sites. Please refer to Gu et al. for a detail description of the RRBS library preparation process [4].

The CpG coverage achieved by RRBS is dependent on the restriction enzymes used to digest the genomic DNA and the sequencing throughput. The use of *MspI* (C|CGG), which is frequently found in CpG islands (CGIs), generates few CG-rich DNA fragments that provide coverage to most of the CGI islands [5]. The coverage of high CG density regions was expanded by the use of a combination of *MspI* and *TaqαI* (T|CGA). This enzyme combination was reported to cover approximately 1.8 million CpGs (sequencing depth of 10 nt), representing approximately 6.6% of the total human CGs [6]. A significant improvement in low density CG region coverage, which includes shore regions and coding sequences (CDS), was achieved by the combined use of *MspI* and *ApeKI* (G|CWGC) [7]. This enzyme combination expanded CpG coverage by approximately 2-fold, while limiting the increase in sequencing cost.

NGS advances have resulted in higher reads and increased sequencing depth, thus allowing novel enzyme combinations to be used. Here, we set out to describe the various enzymes that result in higher genomic and read coverage.

## Methods

MATLAB® 2014a (The MathWorks, Inc., Natick, MA, USA), equipped with a bioinformatics toolbox, was used to create scripts that identify the various parameters measured. Databases were downloaded from the National Center for Biotechnology Information (NCBI) file transfer protocol site, and they were used to ascertain the assembled genomes for *Homo sapiens* (HuRef; annotation release 106), *Rattus novergicus* (NCBI build 4.2), and *Mus musculus* (GRCm38.p2; annotation release 104).

CpGs within fragments were counted from the 5’ and 3’ ends. We established a 40–400 bp fragment cutoff and a sequencing depth of 50 nt to carry out our comparative analysis. For genomic CpG distribution studies, shore regions were up to 2 kb from a CGI, and shelf regions were 2 kb to 4 kb from a CGI. Open sea regions were the genomic CpGs not contained in genes, promoters (2 kb from transcription start site), CGIs, and shore and shelf regions.

**Figure 1:**
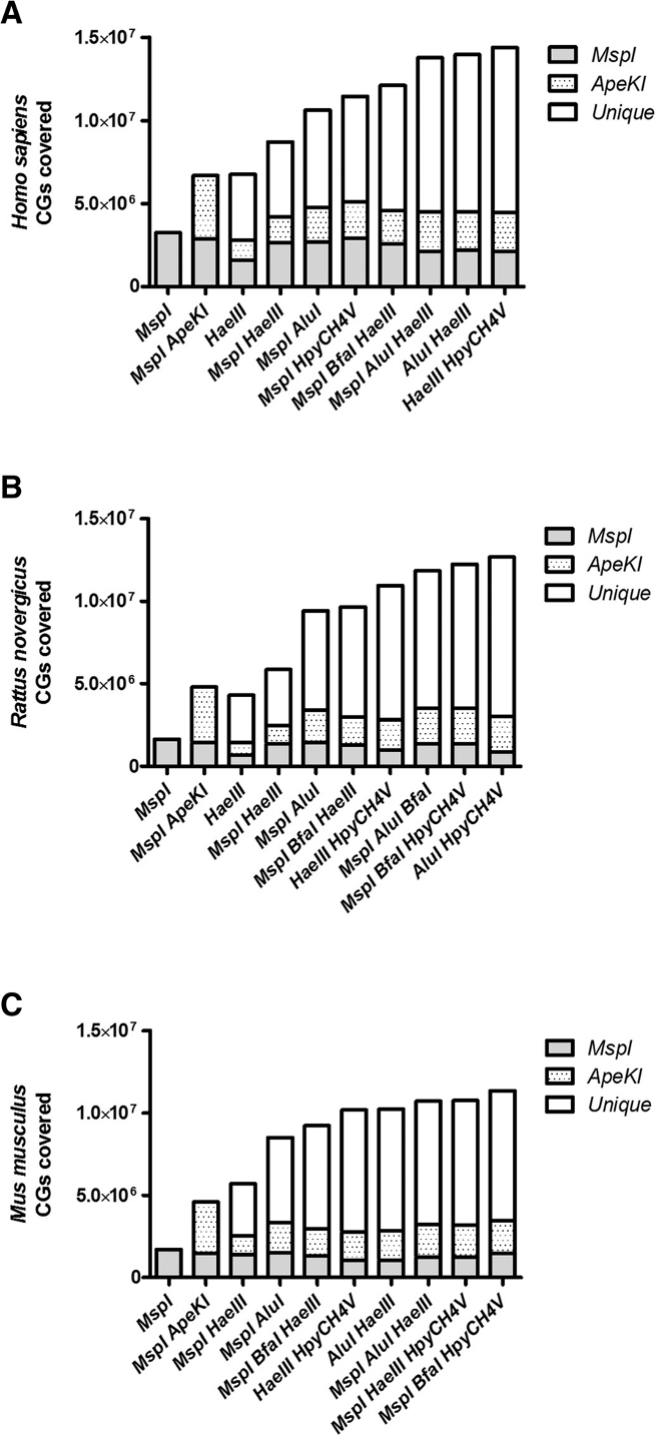
**CpGs covered using selected enzymes with respect to** ***MspI*** **or** ***MspI ApeKI*** **in (A)** ***Homo sapiens*** **, (B)** ***Rattus novergicus*** **, and (C)** ***Mus musculus*** **.**

Synthetic DNA bearing the seven restriction sites approximately every 50 nt was obtained from Integrated DNA Technologies, Inc. (Coralville, IA, USA). Restriction enzymes were obtained from New England Biolabs (Ipswich, MA, USA). The synthetic insert was amplified by PCR using the following cycle parameters: initial 95°C incubation, 30 cycles of 95°C/10 s denaturing, 66°C/10 s annealing, and 72°C/20 s extension, and final 72°C for 7 min incubation. Primer pairs used are shown in Figure 1. Unique band amplification was confirmed by gel electrophoresis and PCR products were purified using the MinElute PCR kit (Qiagen). Restriction digestion of 1 μg of custom-designed DNA was carried out at 37°C for 4 h using 30U *AluI*, 80U *BfaI*, 30U *HaeIII*, 15U *HpyCH4V*, 10U *MluCI*, 10U *MseI*, and 10U *MspI*. Restriction products were size separated in 2% agarose.

## Results and discussion

We began the search for novel enzyme combinations by identifying the 4-nt sequences most frequently found within 50-nt of all genomic CGs. This flanking distance was chosen since it is a common sequencing depth used in NGS (i.e. Illumina HiSeq; Illumina, Inc., San Diego, CA, USA). The user can expect greater CG coverage as the sequencing depth increases. Additional file 1: Tables S1, S2, S3 show the results of the near-neighbor analysis for human, rat, and mouse genomes, respectively. Only the sites for which a restriction enzyme has been described are shown in the analysis. The near-neighbor analysis identified the order of the enzymes that cover the most CGs per organism. Table 1 shows the properties of eleven 4-nt and 5-nt cutting enzymes that are suitable for RRBS use. Seven restriction enzymes (*AluI*, *BfaI*, *HaeIII*, *HpyCH4V*, *MluCI*, *MseI*, and *MspI*) were chosen for our analysis since they share the same reaction conditions, and thus simplify the library preparation process. Moreover, we compared the results of selected enzyme combinations with the outcome of an *MspI* or *MspI ApeKI* combination.

**Table 1:** **Properties of the identified enzymes useful in RRBS**

Enzyme	Consensus sequence	Restriction temp. (°C)	Digestion buffer	Heat inactivation (°C)	Methylation sensitive	Catalog number
*AluI*	AG|CT	37	CutSmart	80	No	R0137S
*BfaI*	C|TAG	37	CutSmart	80	No	R0568S
*HaeIII*	GG|CC	37	CutSmart	80	No	R0108S
*HpyCH4V*	TG|CA	37	CutSmart	65	No	R0620S
*MluCI*	|AATT	37	CutSmart	No	No	R0538S
*MseI*	T|TAA	37	CutSmart	65	No	R0525S
*MspI*	C|CGG	37	CutSmart	No	No	R0106S
*TaqI*	T|CGA	65	CutSmart	80	dam	R0149S
*CviQI*	G|TAC	25	NEB 3.1	No	No	R0639S
*CviAII*	C|ATG	25	CutSmart	65	No	R0640S
*ApeKI*	G|CWGC	75	NEB 3.1	No	Yes	R0643S

We performed in silico restriction digestions with a combination of the seven enzymes selected, and we registered the number of CpGs covered, the number of fragments generated, and identified the genomic coverage (assuming 27 M, 23.9 M, and 21.9 M CpGs for human, rat, and mouse genomes, respectively). In addition, we calculated the CpG/fragment ratio, which represents the density of CGs within a fragment, as generated by a given enzyme. The results show that in the human genome, *MspI* (C|CGG) is the single enzyme that has the highest CpG/fragment ratio, followed by *HaeIII* (GG|CC) and *AluI* (AG|CT). In contrast, *MseI* (T|TAA) and *MluCI* (|AATT) show the lowest CpG/fragment ratio. We also counted the number of fragments that contained no CpGs within the chosen sequencing depth and identified them as CpG-free fragments. We found that, aside from the use of *MspI* alone, the *MspI ApeKI* combination had the least CpG-free fragments (959 K) followed by *HaeIII* (1.5 M) and *MspI HaeIII* (1.7 M). It is important to note that only the use of restriction enzymes that contain CG in their consensus sequence will not generate CpG-free fragments. However, since CG-containing enzymes provide reduced coverage of low density CpG regions, the use of non CG-containing enzymes generates a large number of CpG-free fragments as a byproduct of the increased coverage of low density CpG regions. This has a dramatic impact on the sequencing cost because CpG-free fragments, which are of no value for RRBS, were 31 to 65% of the total fragments generated. Thus, it is up to the end-user to evaluate the benefit of expanding CpG coverage at the expense of increasing sequencing cost.

The individual CpG read coverage was calculated by dividing 150 M (typical reads for the Illumina HiSeq) by the number of fragments generated. The read coverage is critical in selecting an optimal enzyme combination because higher reads have a direct impact on the accuracy of the methylation call. Moreover, the more times a CG is read, it will ultimately improve the subsequent statistics that are applied to find differentially methylated CpGs. We set a read coverage cut-off of 10×, but it is important to note that this threshold is only a baseline used for comparisons and, in practice, is expected to change depending on the mapping rate. We abstained from including a mapping rate into our data because this number varies according to the alignment algorithm used, the size selection of the fragments, and repeat sequences generated for each enzyme combination [8]. However, the user should expect that approximately 70% of the sequences experimentally obtained will be mapped, and thus, result in a proportional decrease of CpG read coverage. Additional file 1: Tables S4, S5, S6 present the full combinatorial analysis, so as to facilitate the choice of the enzyme combination that best suits the investigator’s needs. In Table 2, we present selected enzyme combinations that offer high CpG coverage and at least 10× predicted read coverage. Of note, in the human genome, *HaeIII HpyCH4V* offers the best CpG coverage (53.2%; 10.3× read coverage), but *MspI HypCH4V* offers better read coverage (14.2×) while maintaining high CpG coverage (42.3%). Moreover, recent reports suggest that *MspI* restriction is affected by hydroxymethylation [9], in which case *AluI HaeIII* (CpG coverage, 51.6%; read coverage, 11×) is a suitable substitutions. Higher read coverage can be achieved using *MspI HaeIII* (CpG coverage, 32.2%, read coverage 26.7×) or *HaeIII* (genomic coverage, 25%, read coverage 33.2×). Similar options are also available for the rat and mouse genomes.

**Table 2:** **Summary of enzyme combinations that achieve the best genomic coverage in human, rat, and mouse genomes**

Enzyme combinations	CpGs covered	Fragments generated	Genomic CpG coverage (%)	CpG/fragment ratio	Read coverage
I. *Homo sapiens*					
*HaeIII*	6,755,710	4,521,945	25.0	1.49	33.2×
*MspI HaeIII*	8,703,801	5,613,529	32.2	1.55	26.7×
*MspI AluI*	10,642,461	9,505,806	39.4	1.12	15.8×
*MspI HpyCH4V*	11,443,197	10,567,403	42.3	1.08	14.2×
*MspI BfaI HaeIII*	12,111,370	11,319,689	44.8	1.07	13.3×
*MspI AluI HaeIII*	13,769,398	14,064,014	50.9	0.98	10.7×
*AluI HaeIII*	13,957,636	13,633,940	51.6	1.02	11.0×
*HaeIII HpyCH4V*	14,395,279	14,532,544	53.2	0.99	10.3×
II. *Rattus novergicus*					
*HaeIII*	4,292,555	3,206,578	17.9	1.34	46.8×
*MspI HaeIII*	5,859,114	4,154,939	24.5	1.41	36.1×
*MspI AluI*	9,423,433	8,934,858	39.4	1.05	16.8×
*MspI BfaI HaeIII*	9,643,490	9,579,581	40.3	1.01	15.7×
*HaeIII HpyCH4V*	10,937,751	11,405,956	45.7	0.96	13.2×
*MspI AluI BfaI*	11,837,958	13,122,827	49.5	0.90	11.4×
*MspI BfaI HpyCH4V*	12,246,891	13,543,165	51.2	0.90	11.1×
*AluI HpyCH4V*	12,674,196	14,657,325	53.0	0.86	10.2×
III. *Mus musculus*					
*MspI HaeIII*	5,708,849	4,591,528	26.1	1.24	32.7×
*MspI AluI*	8,524,374	9,393,465	39.0	0.91	16.0×
*MspI BfaI HaeIII*	9,221,025	10,607,497	42.2	0.87	14.1×
*HaeIII HpyCH4V*	10,164,473	12,447,516	46.5	0.82	12.1×
*AluI HaeIII*	10,229,379	12,588,763	46.8	0.81	11.9×
*MspI AluI HaeIII*	10,706,690	13,170,774	49.0	0.81	11.4×
*MspI HaeIII HpyCH4V*	10,759,254	13,095,284	49.2	0.82	11.5×
*MspI BfaI HpyCH4V*	11,347,760	14,601,495	51.9	0.78	10.3×

Calculations assumed a fragment size inclusion of 40–400 bp, a sequencing depth of 50 nt, and a 150 M read NGS throughput.

**Figure 2:**
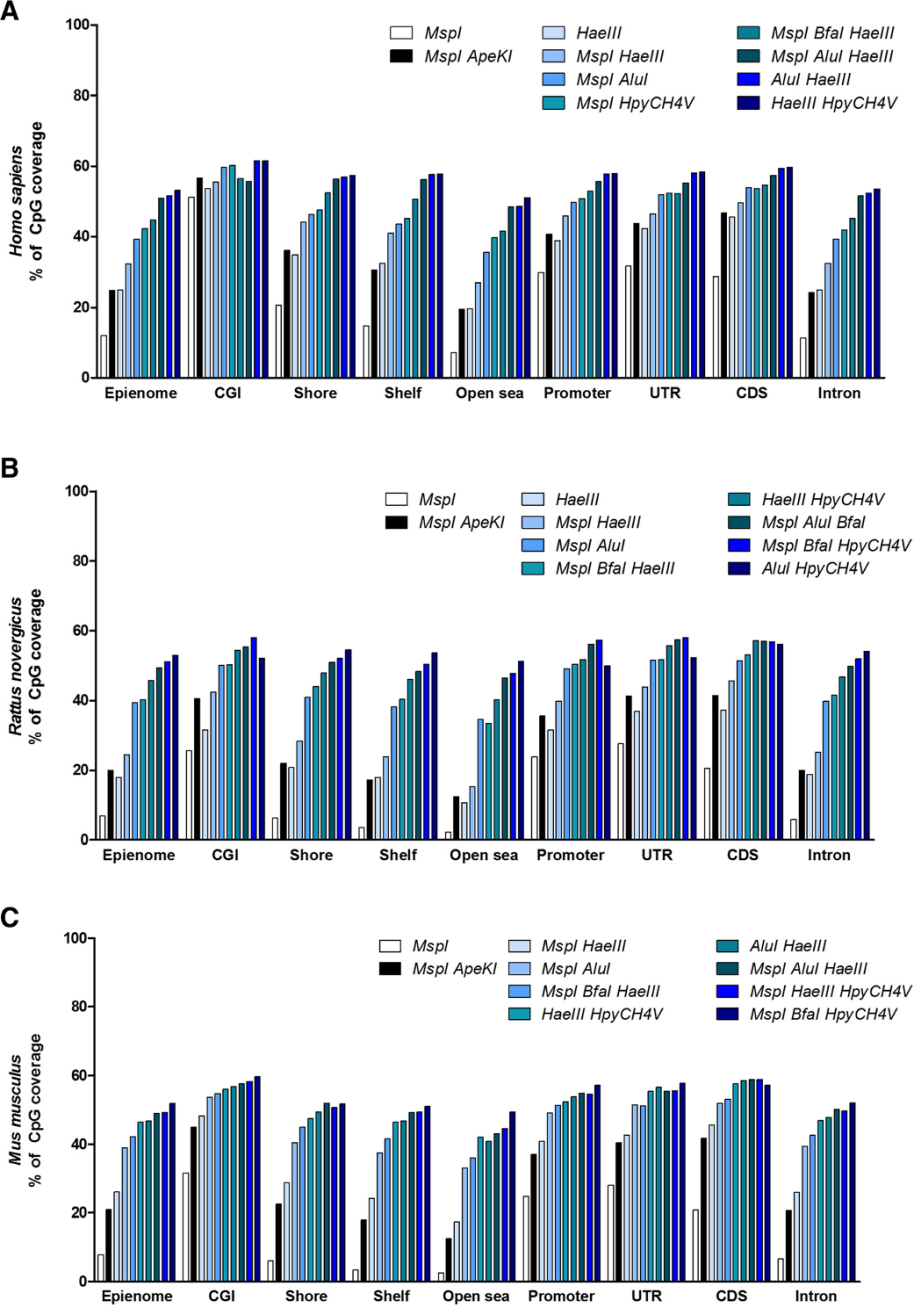
**Genomic distribution of CpGs covered by selected enzymes in (A)** ***Homo sapiens*** **, (B)** ***Rattus novergicus*** **, and (C)** ***Mus musculus*** **.**

We compared the CpGs covered by the enzymes shown in Table 2 with respect to *MspI* or *MspI ApeKI*. Figure 1 shows that, in humans, the use of *ApeKI* doubles the CpGs covered by *MspI*. Similarly, the selected enzyme combinations contained a significant amount of CpGs covered by *MspI* alone or in combination with *ApeKI*, while dramatically increasing the coverage of new CpGs. Interestingly, *HaeIII* showed the least overlapping CpGs when compared to *MspI* or *MspI ApeKI*, but maintained a significant amount of new CpGs covered. Figure 2 shows the genomic distribution of the CpGs covered by the selected enzymes. The results show CGI coverage was similar in all enzymes selected. However, low density CG regions (shore, shelf, open sea, and intron) coverage was significantly improved by the selected enzyme combinations. *HaeIII* showed a similar genomic distribution profile as *MspI ApeKI*. The CpG cover and distribution data suggests that *HaeIII* or *MspI HaeIII* may be used as an alternative to *MspI ApeKI* since they covered a significant amount of new CpGs, while limiting the generation of CpG-free fragments.

We chose to analyze the restriction fragments between 40 – 400 bp to simplify the comparison between enzymes. However, in practice, fragments are size-selected based in their fragment distribution profile. Additional file 2: Figure S1, Additional file 3: Figure S2, Additional file 4: Figure S3 depict the fragment distribution profiles of the enzymes selected in Table 2. The figures show that the majority of the fragments are contained between 40 – 200 bp independently of the enzyme combination used.

**Figure 3:**
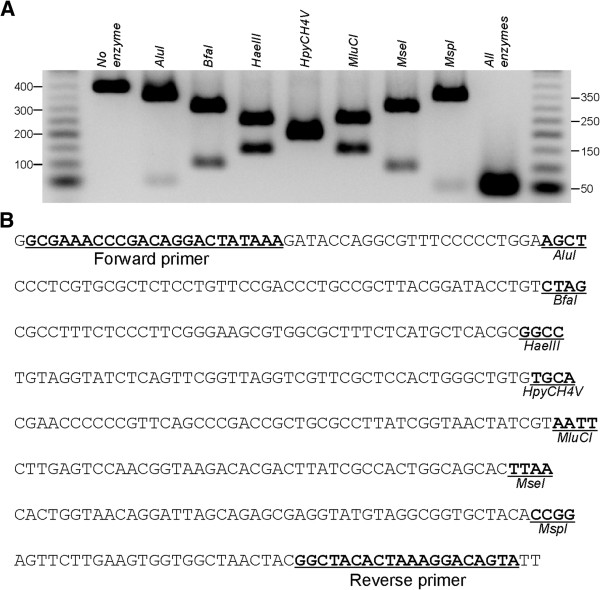
**Restriction digestion assay to evaluate restriction enzyme efficiency and compatibility. (A)** Restriction digestion of DNA bearing the seven restriction sites. **(B)** Sequence used for restriction reaction digestion. Underlined is the primer sequence used to amplify the synthetic insert and the restriction sites.

We performed a restriction assay alone and in combination to assess restriction enzyme efficiency and compatibility. Our initial results using 10U/enzyme for 1 hr at 37°C showed that *BfaI* underperformed in restricting 200 ng of template. Full digestion of 1 μg template was achieved using 80U of *BfaI* for 4 hrs at 37°C in a 50 μl reaction (Figure 3). Here, our aim was to show enzyme compatibility, but the exact conditions for enzyme restriction will have to be determined by the end-user.

## Conclusions

The increase in mass sequencing throughput allows for multiple enzyme combinations to expand RRBS CpG coverage. The use of two or three novel enzyme combinations improves the theoretical CpG coverage to almost half of the human genome. The increased coverage of low density CpG regions generates a significant amount of CpG-free fragments, which considerably increases the sequencing cost. We found that *HaeIII* or *MspI HaeIII* are enzymes that may be used as an alternative to *MspI* or *MspI ApeKI*.

## Electronic supplementary material

Additional file 1: Table S1: Human near-neighbor analysis of 4 nt restriction sites most frequently found within 50 nt of a CpG. In bold are enzymes that may be used for RRBS, and which share similar restriction conditions. Underlined enzymes may need separate restriction reactions. **Table S2.** Rat near-neighbor analysis of 4 nt restriction sites most frequently found within 50 nt of a CpG. In bold are enzymes that may be used for RRBS, and which share similar restriction conditions. Underlined enzymes may need separate restriction reactions. **Table S3.** Mouse near-neighbor analysis of 4 nt restriction sites most frequently found within 50 nt of a CpG. In bold are enzymes that may be used for RRBS, and which share similar restriction conditions. Underlined enzymes may need separate restriction reactions. **Table S4.** Human combinatorial analysis of enzymes that may be used for RRBS. Calculations assumed a fragment size inclusion of 40–400 bp, a sequencing depth of 50 nt, and a 150 M read NGS throughput. **Table S5.** Rat combinatorial analysis of enzymes that may be used for RRBS. Calculations assumed a fragment size inclusion of 40–400 bp, a sequencing depth of 50 nt, and a 150 M read NGS throughput. **Table S6.** Mouse combinatorial analysis of enzymes that may be used for RRBS. Calculations assumed a fragment size inclusion of 40–400 bp, a sequencing depth of 50 nt, and a 150 M read NGS throughput. (DOCX 55 KB)

Additional file 2: Figure S1: Fragment distribution of selected enzymes in *Homo sapiens*. (TIFF 2 MB)

Additional file 3: Figure S2: Fragment distribution of selected enzymes in *Rattus novergicus*. (TIFF 2 MB)

Additional file 4: Figure S3: Fragment distribution of selected enzymes in *Mus musculus*. (TIFF 2 MB)

## Abbreviations

CGI: CpG islands
NGS: Next generation sequencing
RRBS: Reduced representation bisulfite sequencing.
