# Supplementary material for: In silico analysis identifies novel restriction enzyme combinations that expand reduced representation bisulfite sequencing CpG coverage
Source: BMC Res Notes. 2014 Aug 15;7:534. doi: 10.1186/1756-0500-7-534 (PMC4141122; doi:10.1186/1756-0500-7-534)
Supplement: Supplementary file 1 — Additional file 1: Table S1: Human near-neighbor analysis of 4 nt restriction sites most frequently found within 50 nt of a CpG. In bold are enzymes that may be used for RRBS, and which share similar restriction conditions. Underlined enzymes may need separate restriction reactions. Table S2. Rat near-neighbor analysis of 4 nt restriction sites most frequently found within 50 nt of a CpG. In bold are enzymes that may be used for RRBS, and which share similar restriction conditions. Underlined enzymes may need separate restriction reactions. Table S3. Mouse near-neighbor analysis of 4 nt restriction sites most frequently found within 50 nt of a CpG. In bold are enzymes that may be used for RRBS, and which share similar restriction conditions. Underlined enzymes may need separate restriction reactions. Table S4. Human combinatorial analysis of enzymes that may be used for RRBS. Calculations assumed a fragment size inclusion of 40–400 bp, a sequencing depth of 50 nt, and a 150 M read NGS throughput. Table S5. Rat combinatorial analysis of enzymes that may be used for RRBS. Calculations assumed a fragment size inclusion of 40–400 bp, a sequencing depth of 50 nt, and a 150 M read NGS throughput. Table S6. Mouse combinatorial analysis of enzymes that may be used for RRBS. Calculations assumed a fragment size inclusion of 40–400 bp, a sequencing depth of 50 nt, and a 150 M read NGS throughput. (DOCX 55 KB) [file 13104_2014_3055_MOESM1_ESM.docx]

**Additional file**

**Table S1**. Human near-neighbor analysis of 4 nt restriction sites most frequently found within 50 nt of a CpG. In bold are enzymes that may be used for RRBS, and which share similar restriction conditions. Underlined enzymes may need separate restriction reactions.

| Consensus sequence | CpGs near the restriction site | Enzymes |
| --- | --- | --- |
| CCTC | 19,022,521 | *MnlI* |
| AGCC | 15,999,428 | *CviJI / CviKI-1* |
| GGCT | 15,995,599 | *CviJI / CviKI-1* |
| TGCA | 14,726,775 | ***HpyCH4V*** */ CviRI* |
| GGCC | 14,575,062 | ***HaeIII*** */ BshFI / BsnI / BsuRI / CviJI / CviKI-1 / PhoI* |
| AGCT | 13,236,883 | ***AluI*** */ AluBI / CviJI / CviKI-1 / SetI* |
| AATT | 12,297,370 | ***MluCI*** */ Sse9I / TasI / TspEI* |
| CATG | 12,012,006 | *CviAII / FaeI / FaiI / FatI / Hin1II / Hsp92II / NlaIII* |
| ACCT | 11,239,396 | *SetI* |
| AGGT | 11,220,650 | *SetI* |
| TTAA | 9,951,599 | ***MseI*** */ Tru1I / Tru9I* |
| GATC | 8,601,504 | *Asi256I / BfuCI / Bsp143I / BssMI / BstKTI / BstMBI / ChaI / DpnI / DpnII / Kzo9I / MalI / MboI / NdeII / Sau3AI* |
| TATA | 7,991,128 | *FaiI* |
| CCCG | 7,561,101 | *Sth132I* |
| CCGC | 6,827,077 | *AciI / BspACI / SsiI* |
| CATA | 6,542,853 | *FaiI* |
| TATG | 6,540,977 | *FaiI* |
| CCGG | 6,504,186 | ***MspI*** */ BsiSI / HapII / HpaII / Sth302II* |
| GCGC | 6,180,620 | *AspLEI / BstHHI / CfoI / GlaI / HhaI / Hin6I / HinP1I / HspAI* |
| CTAG | 5,799,386 | ***BfaI*** */ FspBI / MaeI / XspI* |
| GTAC | 4,122,728 | *CviQI / AfaI / Csp6I / PabI / RsaI / RsaNI* |
| CGCG | 3,507,189 | *AccII / Bsh1236I / BspFNI / BstFNI / BstUI / FnuDII / MvnI / SelI* |
| ACGT | 2,346,437 | *HpyCH4IV / MaeII / SetI / TaiI* |
| TCGA | 2,096,578 | *TaqI / EsaBC3I* |

**Table S2**. Rat near-neighbor analysis of 4 nt restriction sites most frequently found within 50 nt of a CpG. In bold are enzymes that may be used for RRBS, and which share similar restriction conditions. Underlined enzymes may need separate restriction reactions.

| Consensus sequence | CpG flanking restriction site | Enzymes |
| --- | --- | --- |
| CCTC | 12,543,389 | *MnlI* |
| AGCT | 12,033,609 | ***AluI*** */ AluBI / CviJI / CviKI-1 / SetI* |
| TGCA | 11,166,404 | ***HpyCH4V*** */ CviRI* |
| CATG | 10,650,228 | *CviAII / FaeI / FaiI / FatI / Hin1II / Hsp92II / NlaIII* |
| AGCC | 10,432,476 | *CviJI / CviKI-1* |
| GGCT | 10,423,409 | *CviJI / CviKI-1* |
| TTAA | 9,925,107 | ***MseI*** */ Tru1I / Tru9I* |
| AATT | 9,694,989 | ***MluCI*** */ Sse9I / TasI / TspEI* |
| ACCT | 8,808,029 | *SetI* |
| AGGT | 8,802,663 | *SetI* |
| GGCC | 8,695,795 | ***HaeIII*** */ BshFI / BsnI / BsuRI / CviJI / CviKI-1 / PhoI* |
| TATA | 7,997,151 | *FaiI* |
| TATG | 7,702,370 | *FaiI* |
| CATA | 7,692,970 | *FaiI* |
| CTAG | 6,733,367 | ***BfaI*** */ FspBI / MaeI / XspI* |
| GATC | 6,256,495 | *Asi256I / BfuCI / Bsp143I / BssMI / BstKTI / BstMBI / ChaI / DpnI / DpnII / Kzo9I / MalI / MboI / NdeII / Sau3AI* |
| GTAC | 4,945,105 | *CviQI / AfaI / Csp6I / PabI / RsaI / RsaNI* |
| GCGC | 4,457,268 | *AspLEI / BstHHI / CfoI / GlaI / HhaI / Hin6I / HinP1I / HspAI* |
| CCCG | 3,868,032 | *Sth132I* |
| CCGG | 3,750,764 | ***MspI*** */ BsiSI / HapII / HpaII / Sth302II* |
| CCGC | 3,688,358 | *AciI / BspACI / SsiI* |
| ACGT | 2,485,818 | *HpyCH4IV / MaeII / SetI / TaiI* |
| CGCG | 2,400,079 | *AccII / Bsh1236I / BspFNI / BstFNI / BstUI / FnuDII / MvnI / SelI* |
| TCGA | 1,715,296 | *TaqI* */ EsaBC3I* |

**Table S3**. Mouse near-neighbor analysis of 4 nt restriction sites most frequently found within 50 nt of a CpG. In bold are enzymes that may be used for RRBS, and which share similar restriction conditions. Underlined enzymes may need separate restriction reactions.

| Consensus sequence | CpG flanking restriction site | Enzymes |
| --- | --- | --- |
| CCTC | 11,947,303 | *MnlI* |
| AGCT | 10,750,245 | ***AluI*** */ AluBI / CviJI / CviKI-1 / SetI* |
| TGCA | 10,307,057 | ***HpyCH4V*** */ CviRI* |
| AGCC | 10,202,487 | *CviJI / CviKI-1* |
| GGCT | 10,196,162 | *CviJI / CviKI-1* |
| CATG | 9,675,473 | *CviAII / FaeI / FaiI / FatI / Hin1II / Hsp92II / NlaIII* |
| TTAA | 9,077,040 | ***MseI*** */ Tru1I / Tru9I* |
| AATT | 8,962,194 | ***MluCI*** */ Sse9I / TasI / TspEI* |
| GGCC | 8,955,442 | ***HaeIII*** */ BshFI / BsnI / BsuRI / CviJI / CviKI-1 / PhoI* |
| ACCT | 8,268,493 | *SetI* |
| AGGT | 8,261,751 | *SetI* |
| TATA | 7,284,676 | *FaiI* |
| CATA | 7,133,533 | *FaiI* |
| TATG | 7,130,440 | *FaiI* |
| CTAG | 6,337,017 | ***BfaI*** */ FspBI / MaeI / XspI* |
| GATC | 5,778,924 | *Asi256I / BfuCI / Bsp143I / BssMI / BstKTI / BstMBI / ChaI / DpnI / DpnII / Kzo9I / MalI / MboI / NdeII / Sau3AI* |
| GTAC | 4,572,698 | *CviQI / AfaI / Csp6I / PabI / RsaI / RsaNI* |
| CCCG | 4,057,537 | *Sth132I* |
| GCGC | 4,035,125 | *AspLEI / BstHHI / CfoI / GlaI / HhaI / Hin6I / HinP1I / HspAI* |
| CCGC | 3,899,648 | *AciI / BspACI / SsiI* |
| CCGG | 3,882,680 | ***MspI*** */ BsiSI / HapII / HpaII / Sth302II* |
| CGCG | 2,387,516 | *AccII / Bsh1236I / BspFNI / BstFNI / BstUI / FnuDII / MvnI / SelI* |
| ACGT | 1,986,050 | *HpyCH4IV / MaeII / SetI / TaiI* |
| TCGA | 1,577,922 | *TaqI / EsaBC3I* |

**Table S4**. Human combinatorial analysis of enzymes that may be used for RRBS. Calculations assumed a fragment size inclusion of 40–400 bp, a sequencing depth of 50 nt, and a 150M read NGS throughput.

| Enzyme combinations | CpGs covered | Fragments generated | Genomic CpG coverage (%) | CpG / fragment ratio | Read coverage | CpG-free fragments |
| --- | --- | --- | --- | --- | --- | --- |
| *MspI* | 3,249,789 | 824,361 | 12.0 | 3.94 | 182.0 | 0 |
| *MspI ApeKI* | 6,697,597 | 3,612,028 | 24.8 | 1.85 | 41.5 | 959,656 |
|  |  |  |  |  |  |  |
| *BfaI* | 2,913,117 | 4,192,800 | 10.8 | 0.69 | 35.8 | 2,356,945 |
| *MseI* | 5,577,402 | 11,451,948 | 20.6 | 0.49 | 13.1 | 7,461,791 |
| *HaeIII* | 6,755,710 | 4,521,945 | 25.0 | 1.49 | 33.2 | 1,561,734 |
| *MspI BfaI* | 7,559,632 | 5,977,553 | 28.0 | 1.26 | 25.1 | 2,570,348 |
| *MluCI* | 7,619,418 | 13,337,039 | 28.2 | 0.57 | 11.2 | 8,261,828 |
| *AluI* | 7,871,789 | 8,277,056 | 29.1 | 0.95 | 18.1 | 3,994,034 |
| *HpyCH4V* | 7,971,346 | 9,117,716 | 29.5 | 0.87 | 16.5 | 4,619,956 |
| *MspI HaeIII* | 8,703,801 | 5,613,529 | 32.2 | 1.55 | 26.7 | 1,756,397 |
| *MseI BfaI* | 9,145,019 | 16,050,476 | 33.8 | 0.57 | 9.3 | 9,978,481 |
| *MspI MseI* | 10,441,451 | 13,260,012 | 38.6 | 0.79 | 11.3 | 7,666,306 |
| *BfaI HaeIII* | 10,568,928 | 10,315,412 | 39.1 | 1.02 | 14.5 | 4,871,613 |
| *MspI AluI* | 10,642,461 | 9,505,806 | 39.4 | 1.12 | 15.8 | 4,208,023 |
| *MseI MluCI* | 10,756,622 | 18,213,969 | 39.8 | 0.59 | 8.2 | 11,209,652 |
| *BfaI MluCI* | 10,936,796 | 17,578,141 | 40.4 | 0.62 | 8.5 | 10,590,940 |
| *AluI BfaI* | 11,304,810 | 13,247,707 | 41.8 | 0.85 | 11.3 | 6,872,554 |
| *MspI HpyCH4V* | 11,443,197 | 10,567,403 | 42.3 | 1.08 | 14.2 | 4,846,543 |
| *BfaI HpyCH4V* | 11,663,721 | 14,610,600 | 43.1 | 0.80 | 10.3 | 7,855,779 |
| *MspI MluCI* | 11,824,198 | 14,896,548 | 43.7 | 0.79 | 10.1 | 8,464,123 |
| *MspI BfaI HaeIII* | 12,111,370 | 11,319,689 | 44.8 | 1.07 | 13.3 | 5,097,412 |
| *MseI BfaI MluCI* | 13,118,710 | 20,792,308 | 48.5 | 0.63 | 7.2 | 12,545,446 |
| *MseI HaeIII* | 13,234,671 | 17,273,630 | 48.9 | 0.77 | 8.7 | 9,768,268 |
| *MseI HpyCH4V* | 13,273,040 | 19,331,772 | 49.1 | 0.69 | 7.8 | 11,343,647 |
| *MspI AluI BfaI* | 13,298,418 | 14,237,864 | 49.2 | 0.93 | 10.5 | 7,093,715 |
| *MspI MseI BfaI* | 13,381,638 | 17,642,735 | 49.5 | 0.76 | 8.5 | 10,187,895 |
| *AluI MseI* | 13,491,157 | 19,059,071 | 49.9 | 0.71 | 7.9 | 11,035,144 |
| *AluI MluCI* | 13,647,040 | 19,769,939 | 50.5 | 0.69 | 7.6 | 11,501,773 |
| *MspI AluI HaeIII* | 13,769,398 | 14,064,014 | 50.9 | 0.98 | 10.7 | 6,600,825 |
| *AluI HpyCH4V* | 13,887,052 | 16,811,312 | 51.4 | 0.83 | 8.9 | 8,853,737 |
| *AluI HaeIII* | 13,957,636 | 13,633,940 | 51.6 | 1.02 | 11.0 | 6,379,685 |
| *MspI BfaI HpyCH4V* | 14,203,585 | 15,765,913 | 52.5 | 0.90 | 9.5 | 8,086,948 |
| *MluCI HpyCH4V* | 14,378,574 | 20,348,757 | 53.2 | 0.71 | 7.4 | 11,769,835 |
| *HaeIII HpyCH4V* | 14,395,279 | 14,532,544 | 53.2 | 0.99 | 10.3 | 6,981,358 |
| *MspI MseI MluCI* | 14,406,345 | 19,537,412 | 53.3 | 0.74 | 7.7 | 11,406,838 |
| *MspI BfaI MluCI* | 14,456,418 | 18,912,509 | 53.5 | 0.76 | 7.9 | 10,800,234 |
| *MspI HaeIII HpyCH4V* | 14,661,044 | 15,141,355 | 54.2 | 0.97 | 9.9 | 7,212,272 |
| *MspI MseI HaeIII* | 14,704,021 | 18,189,047 | 54.4 | 0.81 | 8.2 | 9,980,079 |
| *MluCI HaeIII* | 14,798,674 | 18,701,616 | 54.7 | 0.79 | 8.0 | 10,426,140 |
| *MspI AluI HpyCH4V* | 14,892,064 | 17,451,318 | 55.1 | 0.85 | 8.6 | 9,070,307 |
| *MseI BfaI HpyCH4V* | 15,155,332 | 21,926,878 | 56.1 | 0.69 | 6.8 | 12,890,486 |
| *AluI MseI MluCI* | 15,224,642 | 22,329,705 | 56.3 | 0.68 | 6.7 | 13,150,320 |
| *MseI BfaI HaeIII* | 15,226,952 | 20,526,223 | 56.3 | 0.74 | 7.3 | 11,746,955 |
| *AluI MseI BfaI* | 15,256,401 | 21,447,745 | 56.4 | 0.71 | 7.0 | 12,434,336 |
| *AluI BfaI MluCI* | 15,264,713 | 21,915,729 | 56.5 | 0.70 | 6.8 | 12,758,895 |
| *MspI AluI MseI* | 15,273,834 | 19,898,531 | 56.5 | 0.77 | 7.5 | 11,239,387 |
| *MspI AluI MluCI* | 15,439,458 | 20,569,696 | 57.1 | 0.75 | 7.3 | 11,703,079 |
| *AluI BfaI HpyCH4V* | 15,590,037 | 19,870,576 | 57.7 | 0.78 | 7.5 | 10,833,288 |
| *MspI MluCI HaeIII* | 15,592,116 | 19,392,297 | 57.7 | 0.80 | 7.7 | 10,639,808 |
| *MspI MseI HpyCH4V* | 15,681,281 | 20,373,546 | 58.0 | 0.77 | 7.4 | 11,556,633 |
| *AluI BfaI HaeIII* | 15,785,993 | 17,419,414 | 58.4 | 0.91 | 8.6 | 8,828,003 |
| *MseI MluCI HpyCH4V* | 15,796,875 | 22,726,789 | 58.4 | 0.70 | 6.6 | 13,330,291 |
| *BfaI MluCI HpyCH4V* | 16,047,765 | 22,727,851 | 59.4 | 0.71 | 6.6 | 13,213,297 |
| *BfaI HaeIII HpyCH4V* | 16,228,147 | 18,634,868 | 60.0 | 0.87 | 8.0 | 9,695,991 |
| *MspI MluCI HpyCH4V* | 16,312,230 | 21,243,436 | 60.3 | 0.77 | 7.1 | 11,981,608 |
| *AluI MluCI HpyCH4V* | 16,355,153 | 23,459,182 | 60.5 | 0.70 | 6.4 | 13,627,675 |
| *BfaI MluCI HaeIII* | 16,479,712 | 21,620,264 | 61.0 | 0.76 | 6.9 | 12,245,246 |
| *AluI MseI HpyCH4V* | 16,564,053 | 23,194,736 | 61.3 | 0.71 | 6.5 | 13,411,776 |
| *MseI MluCI HaeIII* | 16,611,424 | 22,311,765 | 61.4 | 0.74 | 6.7 | 12,819,043 |
| *AluI HaeIII HpyCH4V* | 17,079,748 | 20,174,999 | 63.2 | 0.85 | 7.4 | 10,559,128 |
| *AluI MluCI HaeIII* | 17,369,221 | 22,925,547 | 64.2 | 0.76 | 6.5 | 12,943,779 |
| *AluI MseI HaeIII* | 17,508,662 | 22,534,020 | 64.8 | 0.78 | 6.7 | 12,602,233 |
| *MseI HaeIII HpyCH4V* | 17,520,404 | 22,798,896 | 64.8 | 0.77 | 6.6 | 12,852,284 |
| *MluCI HaeIII HpyCH4V* | 18,224,285 | 23,544,108 | 67.4 | 0.77 | 6.4 | 13,225,527 |

**Table S5**. Rat combinatorial analysis of enzymes that may be used for RRBS. Calculations assumed a fragment size inclusion of 40–400 bp, a sequencing depth of 50 nt, and a 150M read NGS throughput.

| Enzyme combinations | CpGs covered | Fragments generated | Genomic CpG coverage (%) | CpG / fragment ratio | Read coverage | CpG-free fragments |
| --- | --- | --- | --- | --- | --- | --- |
| *MspI* | 1,649,617 | 487,304 | 6.9 | 3.39 | 307.8 | 0 |
| *MspI ApeKI* | 4,788,201 | 2,875,056 | 20.0 | 1.67 | 52.2 | 729,555 |
|  |  |  |  |  |  |  |
| *BfaI* | 3,606,311 | 4,246,308 | 15.1 | 0.85 | 35.3 | 2,098,934 |
| *HaeIII* | 4,292,555 | 3,206,578 | 17.9 | 1.34 | 46.8 | 1,149,390 |
| *MluCI* | 5,495,892 | 9,270,888 | 23.0 | 0.59 | 16.2 | 5,504,195 |
| *MseI* | 5,718,043 | 9,008,401 | 23.9 | 0.63 | 16.7 | 5,197,158 |
| *MspI HaeIII* | 5,859,114 | 4,154,939 | 24.5 | 1.41 | 36.1 | 1,314,061 |
| *MspI BfaI* | 6,410,634 | 5,583,894 | 26.8 | 1.15 | 26.9 | 2,295,906 |
| *HpyCH4V* | 6,458,561 | 7,111,014 | 27.0 | 0.91 | 21.1 | 3,396,720 |
| *AluI* | 7,377,048 | 7,792,454 | 30.8 | 0.95 | 19.2 | 3,542,803 |
| *MspI MluCI* | 8,422,061 | 10,609,858 | 35.2 | 0.79 | 14.1 | 5,702,192 |
| *BfaI HaeIII* | 8,505,003 | 8,737,147 | 35.5 | 0.97 | 17.2 | 4,018,600 |
| *MspI MseI* | 8,525,092 | 10,322,833 | 35.6 | 0.83 | 14.5 | 5,397,456 |
| *MspI HpyCH4V* | 8,646,386 | 8,243,716 | 36.1 | 1.05 | 18.2 | 3,594,027 |
| *MseI MluCI* | 9,318,715 | 14,486,711 | 38.9 | 0.64 | 10.4 | 8,355,273 |
| *MspI AluI* | 9,423,433 | 8,934,858 | 39.4 | 1.05 | 16.8 | 3,754,793 |
| *MspI BfaI HaeIII* | 9,643,490 | 9,579,581 | 40.3 | 1.01 | 15.7 | 4,202,899 |
| *BfaI MluCI* | 9,833,956 | 14,212,376 | 41.1 | 0.69 | 10.6 | 7,915,101 |
| *MseI BfaI* | 9,995,603 | 14,070,177 | 41.8 | 0.71 | 10.7 | 7,757,251 |
| *AluI BfaI* | 10,434,152 | 12,258,168 | 43.6 | 0.85 | 12.2 | 6,039,212 |
| *AluI HaeIII* | 10,855,432 | 11,639,907 | 45.4 | 0.93 | 12.9 | 5,403,092 |
| *MluCI HaeIII* | 10,895,535 | 13,830,234 | 45.5 | 0.79 | 10.8 | 7,347,326 |
| *MseI HaeIII* | 10,901,714 | 13,524,043 | 45.6 | 0.81 | 11.1 | 7,077,343 |
| *HaeIII HpyCH4V* | 10,937,751 | 11,405,956 | 45.7 | 0.96 | 13.2 | 5,275,191 |
| *BfaI HpyCH4V* | 10,950,074 | 12,710,297 | 45.8 | 0.86 | 11.8 | 6,301,368 |
| *MspI AluI HaeIII* | 11,481,362 | 12,305,340 | 48.0 | 0.93 | 12.2 | 5,601,433 |
| *MspI HaeIII HpyCH4V* | 11,537,539 | 12,055,881 | 48.2 | 0.96 | 12.4 | 5,475,805 |
| *MluCI HpyCH4V* | 11,689,741 | 15,871,281 | 48.8 | 0.74 | 9.5 | 8,613,805 |
| *MspI MseI MluCI* | 11,737,658 | 15,607,513 | 49.0 | 0.75 | 9.6 | 8,558,435 |
| *MspI AluI BfaI* | 11,837,958 | 13,122,827 | 49.5 | 0.90 | 11.4 | 6,243,890 |
| *MseI HpyCH4V* | 11,842,504 | 15,876,518 | 49.5 | 0.75 | 9.4 | 8,574,036 |
| *MspI MseI HaeIII* | 11,921,176 | 14,309,119 | 49.8 | 0.83 | 10.5 | 7,283,040 |
| *MspI MluCI HaeIII* | 11,969,561 | 14,632,371 | 50.0 | 0.82 | 10.3 | 7,551,694 |
| *MspI BfaI MluCI* | 12,003,646 | 15,282,053 | 50.2 | 0.79 | 9.8 | 8,125,276 |
| *MspI MseI BfaI* | 12,052,375 | 15,106,425 | 50.4 | 0.80 | 9.9 | 7,949,997 |
| *MspI BfaI HpyCH4V* | 12,246,891 | 13,543,165 | 51.2 | 0.90 | 11.1 | 6,505,533 |
| *MseI BfaI MluCI* | 12,488,691 | 17,925,578 | 52.2 | 0.70 | 8.4 | 10,021,032 |
| *AluI MluCI* | 12,578,250 | 16,746,319 | 52.6 | 0.75 | 9.0 | 9,009,237 |
| *AluI HpyCH4V* | 12,674,196 | 14,657,325 | 53.0 | 0.86 | 10.2 | 7,255,981 |
| *AluI MseI* | 12,708,434 | 16,510,974 | 53.1 | 0.77 | 9.1 | 8,714,855 |
| *AluI BfaI HaeIII* | 12,936,313 | 15,156,496 | 54.1 | 0.85 | 9.9 | 7,485,672 |
| *MspI MluCI HpyCH4V* | 13,197,436 | 16,710,667 | 55.1 | 0.79 | 9.0 | 8,813,574 |
| *MspI MseI HpyCH4V* | 13,254,511 | 16,683,715 | 55.4 | 0.79 | 9.0 | 8,778,954 |
| *MseI BfaI HaeIII* | 13,335,104 | 17,179,313 | 55.7 | 0.78 | 8.7 | 9,155,451 |
| *BfaI MluCI HaeIII* | 13,356,882 | 17,360,021 | 55.8 | 0.77 | 8.6 | 9,282,691 |
| *MspI AluI HpyCH4V* | 13,445,617 | 15,316,632 | 56.2 | 0.88 | 9.8 | 7,461,086 |
| *MseI MluCI HaeIII* | 13,456,903 | 17,885,303 | 56.2 | 0.75 | 8.4 | 9,724,710 |
| *BfaI HaeIII HpyCH4V* | 13,506,319 | 15,627,402 | 56.4 | 0.86 | 9.6 | 7,728,215 |
| *MseI MluCI HpyCH4V* | 13,652,701 | 18,967,311 | 57.0 | 0.72 | 7.9 | 10,491,472 |
| *MspI AluI MluCI* | 13,850,245 | 17,536,436 | 57.9 | 0.79 | 8.6 | 9,216,285 |
| *MspI AluI MseI* | 13,928,011 | 17,277,336 | 58.2 | 0.81 | 8.7 | 8,927,844 |
| *AluI BfaI MluCI* | 14,033,464 | 19,031,529 | 58.6 | 0.74 | 7.9 | 10,352,116 |
| *AluI MseI BfaI* | 14,131,578 | 18,932,232 | 59.0 | 0.75 | 7.9 | 10,213,121 |
| *AluI HaeIII HpyCH4V* | 14,140,442 | 16,975,536 | 59.1 | 0.83 | 8.8 | 8,562,219 |
| *AluI BfaI HpyCH4V* | 14,209,683 | 17,564,061 | 59.4 | 0.81 | 8.5 | 9,067,266 |
| *BfaI MluCI HpyCH4V* | 14,359,112 | 19,091,687 | 60.0 | 0.75 | 7.9 | 10,322,470 |
| *AluI MseI MluCI* | 14,417,570 | 19,621,749 | 60.2 | 0.73 | 7.6 | 10,731,579 |
| *MseI BfaI HpyCH4V* | 14,418,230 | 19,139,287 | 60.2 | 0.75 | 7.8 | 10,375,154 |
| *AluI MluCI HaeIII* | 14,564,576 | 19,051,805 | 60.9 | 0.76 | 7.9 | 10,199,606 |
| *AluI MseI HaeIII* | 14,597,890 | 18,821,257 | 61.0 | 0.78 | 8.0 | 9,942,626 |
| *MseI HaeIII HpyCH4V* | 14,657,853 | 18,634,203 | 61.2 | 0.79 | 8.0 | 9,841,517 |
| *MluCI HaeIII HpyCH4V* | 14,659,804 | 18,688,222 | 61.3 | 0.78 | 8.0 | 9,883,486 |
| *AluI MluCI HpyCH4V* | 15,326,562 | 20,211,361 | 64.0 | 0.76 | 7.4 | 10,912,914 |
| *AluI MseI HpyCH4V* | 15,396,538 | 20,152,388 | 64.3 | 0.76 | 7.4 | 10,809,286 |

**Table S6**. Mouse combinatorial analysis of enzymes that may be used for RRBS. Calculations assumed a fragment size inclusion of 40–400 bp, a sequencing depth of 50 nt, and a 150M read NGS throughput.

| Enzyme combinations | CpGs covered | Fragments generated | Genomic CpG coverage (%) | CpG / fragment ratio | Read coverage | CpG-free fragments |
| --- | --- | --- | --- | --- | --- | --- |
| *MspI* | 1,704,894 | 487,051 | 7.8 | 3.50 | 308.0 | 0 |
| *MspI ApeKI* | 4,592,211 | 2,997,461 | 21.0 | 1.53 | 50.0 | 912,898 |
|  |  |  |  |  |  |  |
| *BfaI* | 3,405,646 | 4,897,387 | 15.6 | 0.70 | 30.6 | 2,689,158 |
| *HaeIII* | 4,376,203 | 3,709,433 | 20.0 | 1.18 | 40.4 | 1,574,074 |
| *MluCI* | 5,177,016 | 10,194,506 | 23.7 | 0.51 | 14.7 | 6,528,035 |
| *MseI* | 5,236,435 | 9,629,198 | 23.9 | 0.54 | 15.6 | 6,029,987 |
| *MspI HaeIII* | 5,708,849 | 4,591,528 | 26.1 | 1.24 | 32.7 | 1,766,381 |
| *HpyCH4V* | 5,992,526 | 7,817,635 | 27.4 | 0.77 | 19.2 | 4,111,363 |
| *MspI BfaI* | 6,055,897 | 6,147,138 | 27.7 | 0.99 | 24.4 | 2,926,330 |
| *AluI* | 6,535,203 | 8,364,804 | 29.9 | 0.78 | 17.9 | 4,382,373 |
| *MspI MluCI* | 7,946,196 | 11,505,377 | 36.3 | 0.69 | 13.0 | 6,753,016 |
| *MspI MseI* | 7,958,246 | 10,929,945 | 36.4 | 0.73 | 13.7 | 6,248,975 |
| *MspI HpyCH4V* | 8,312,086 | 8,993,919 | 38.0 | 0.92 | 16.7 | 4,344,152 |
| *BfaI HaeIII* | 8,326,311 | 9,840,155 | 38.1 | 0.85 | 15.2 | 5,081,625 |
| *MspI AluI* | 8,524,374 | 9,393,465 | 39.0 | 0.91 | 16.0 | 4,612,823 |
| *MseI MluCI* | 8,619,151 | 15,546,065 | 39.4 | 0.55 | 9.6 | 9,677,797 |
| *BfaI MluCI* | 9,097,542 | 15,620,886 | 41.6 | 0.58 | 9.6 | 9,507,880 |
| *MseI BfaI* | 9,173,893 | 15,237,869 | 42.0 | 0.60 | 9.8 | 9,139,775 |
| *MspI BfaI HaeIII* | 9,221,025 | 10,607,497 | 42.2 | 0.87 | 14.1 | 5,323,382 |
| *AluI BfaI* | 9,501,298 | 13,027,916 | 43.4 | 0.73 | 11.5 | 7,157,254 |
| *BfaI HpyCH4V* | 9,764,084 | 13,725,128 | 44.7 | 0.71 | 10.9 | 7,580,327 |
| *HaeIII HpyCH4V* | 10,164,473 | 12,447,516 | 46.5 | 0.82 | 12.1 | 6,499,487 |
| *AluI HaeIII* | 10,229,379 | 12,588,763 | 46.8 | 0.81 | 11.9 | 6,610,280 |
| *MseI HaeIII* | 10,292,257 | 14,747,106 | 47.1 | 0.70 | 10.2 | 8,509,354 |
| *MluCI HaeIII* | 10,361,141 | 15,240,880 | 47.4 | 0.68 | 9.8 | 8,896,279 |
| *MspI AluI HaeIII* | 10,706,690 | 13,170,774 | 49.0 | 0.81 | 11.4 | 6,836,742 |
| *MluCI HpyCH4V* | 10,740,690 | 17,294,491 | 49.1 | 0.62 | 8.7 | 10,276,855 |
| *MspI HaeIII HpyCH4V* | 10,759,254 | 13,095,284 | 49.2 | 0.82 | 11.5 | 6,734,767 |
| *MspI MseI MluCI* | 10,843,718 | 16,616,421 | 49.6 | 0.65 | 9.0 | 9,902,729 |
| *MspI AluI BfaI* | 10,963,633 | 13,868,373 | 50.1 | 0.79 | 10.8 | 7,387,139 |
| *MseI HpyCH4V* | 10,969,905 | 17,175,678 | 50.2 | 0.64 | 8.7 | 10,067,324 |
| *MspI MseI HaeIII* | 11,032,574 | 15,458,863 | 50.5 | 0.71 | 9.7 | 8,741,290 |
| *MspI BfaI MluCI* | 11,140,854 | 16,603,408 | 50.9 | 0.67 | 9.0 | 9,749,055 |
| *MspI MseI BfaI* | 11,144,362 | 16,204,294 | 51.0 | 0.69 | 9.3 | 9,375,434 |
| *MspI MluCI HaeIII* | 11,160,916 | 15,949,659 | 51.0 | 0.70 | 9.4 | 9,128,946 |
| *AluI MseI* | 11,288,699 | 17,509,958 | 51.6 | 0.64 | 8.6 | 10,276,382 |
| *AluI MluCI* | 11,339,461 | 18,042,308 | 51.9 | 0.63 | 8.3 | 10,709,516 |
| *MspI BfaI HpyCH4V* | 11,347,760 | 14,601,495 | 51.9 | 0.78 | 10.3 | 7,822,398 |
| *AluI HpyCH4V* | 11,416,799 | 15,746,434 | 52.2 | 0.73 | 9.5 | 8,654,548 |
| *MseI BfaI MluCI* | 11,446,335 | 19,305,735 | 52.3 | 0.59 | 7.8 | 11,727,171 |
| *AluI BfaI HaeIII* | 12,029,788 | 16,182,962 | 55.0 | 0.74 | 9.3 | 8,922,750 |
| *MspI MluCI HpyCH4V* | 12,331,442 | 18,146,082 | 56.4 | 0.68 | 8.3 | 10,514,915 |
| *MspI AluI HpyCH4V* | 12,385,042 | 16,392,854 | 56.6 | 0.76 | 9.2 | 8,882,758 |
| *BfaI HaeIII HpyCH4V* | 12,463,319 | 16,921,563 | 57.0 | 0.74 | 8.9 | 9,333,207 |
| *MspI MseI HpyCH4V* | 12,466,968 | 17,994,138 | 57.0 | 0.69 | 8.3 | 10,302,078 |
| *MspI AluI MseI* | 12,478,760 | 18,181,930 | 57.1 | 0.69 | 8.2 | 10,489,903 |
| *MseI BfaI HaeIII* | 12,532,138 | 18,681,380 | 57.3 | 0.67 | 8.0 | 10,927,182 |
| *MspI AluI MluCI* | 12,587,893 | 18,732,068 | 57.6 | 0.67 | 8.0 | 10,937,163 |
| *MseI MluCI HpyCH4V* | 12,592,292 | 20,384,595 | 57.6 | 0.62 | 7.4 | 12,199,838 |
| *BfaI MluCI HaeIII* | 12,615,857 | 19,015,637 | 57.7 | 0.66 | 7.9 | 11,186,933 |
| *MseI MluCI HaeIII* | 12,619,688 | 19,409,507 | 57.7 | 0.65 | 7.7 | 11,530,492 |
| *AluI MseI BfaI* | 12,813,567 | 19,972,666 | 58.6 | 0.64 | 7.5 | 11,805,322 |
| *BfaI MluCI HpyCH4V* | 12,818,914 | 20,470,036 | 58.6 | 0.63 | 7.3 | 12,199,046 |
| *AluI BfaI MluCI* | 12,883,490 | 20,373,154 | 58.9 | 0.63 | 7.4 | 12,127,672 |
| *AluI BfaI HpyCH4V* | 12,894,033 | 18,618,808 | 59.0 | 0.69 | 8.1 | 10,552,275 |
| *MseI BfaI HpyCH4V* | 12,946,436 | 20,382,752 | 59.2 | 0.64 | 7.4 | 12,045,970 |
| *AluI MseI MluCI* | 12,984,981 | 20,921,823 | 59.4 | 0.62 | 7.2 | 12,537,861 |
| *AluI HaeIII HpyCH4V* | 13,142,074 | 18,268,455 | 60.1 | 0.72 | 8.2 | 10,190,801 |
| *AluI MseI HaeIII* | 13,451,617 | 20,178,949 | 61.5 | 0.67 | 7.4 | 11,814,977 |
| *MluCI HaeIII HpyCH4V* | 13,453,956 | 20,265,726 | 61.5 | 0.66 | 7.4 | 11,872,527 |
| *MseI HaeIII HpyCH4V* | 13,514,144 | 20,161,663 | 61.8 | 0.67 | 7.4 | 11,743,816 |
| *AluI MluCI HaeIII* | 13,599,192 | 20,611,546 | 62.2 | 0.66 | 7.3 | 12,119,391 |
| *AluI MluCI HpyCH4V* | 13,789,604 | 21,655,124 | 63.1 | 0.64 | 6.9 | 12,843,730 |
| *AluI MseI HpyCH4V* | 13,831,601 | 21,444,287 | 63.3 | 0.65 | 7.0 | 12,626,247 |
