# Supplementary figures and images for: In silico analysis identifies novel restriction enzyme combinations that expand reduced representation bisulfite sequencing CpG coverage
Source: BMC Res Notes. 2014 Aug 15;7:534. doi: 10.1186/1756-0500-7-534 (PMC4141122; doi:10.1186/1756-0500-7-534)

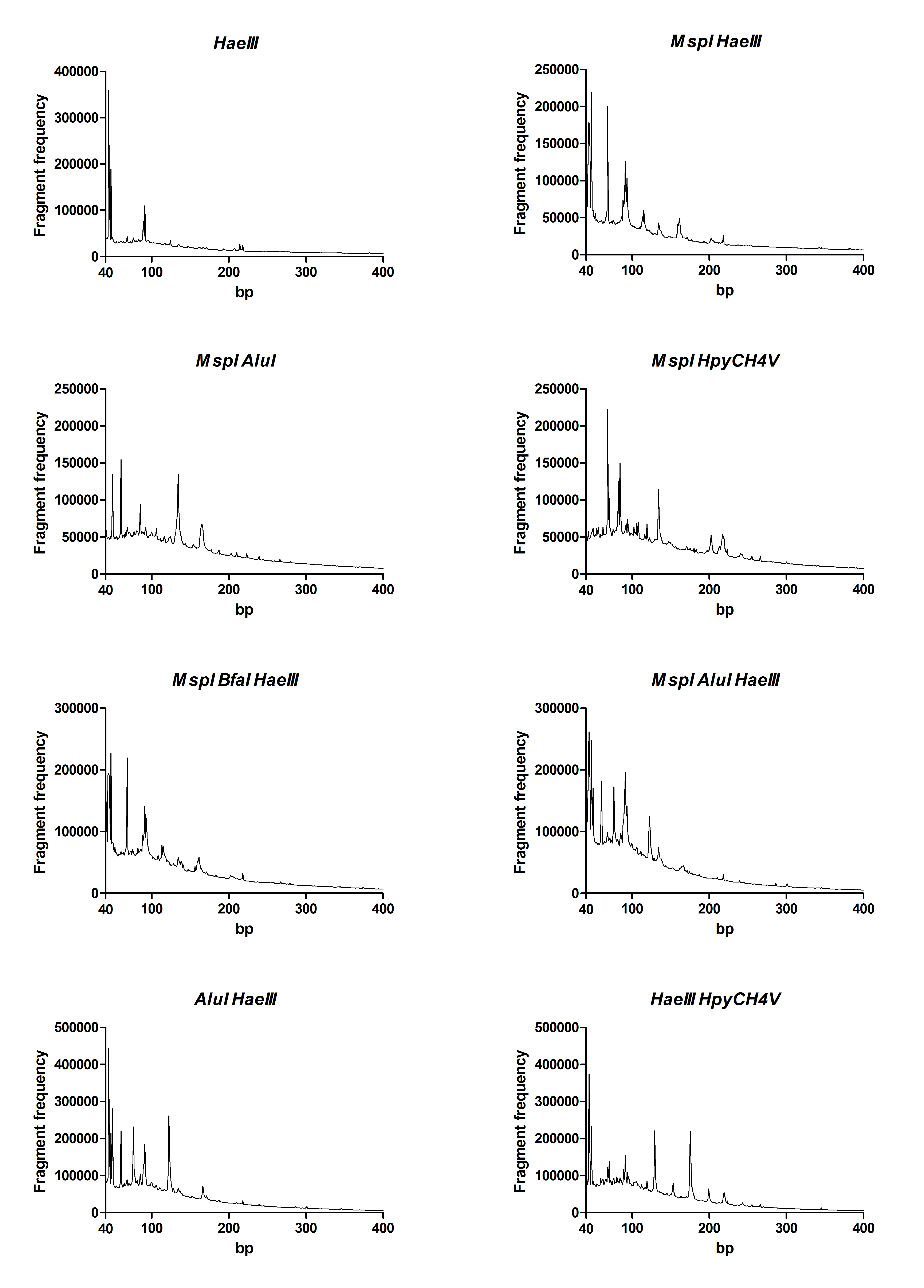

Supplement: Supplementary file 2 — Additional file 2: Figure S1: Fragment distribution of selected enzymes in Homo sapiens. (TIFF 2 MB) [file 13104_2014_3055_MOESM2_ESM.tiff]

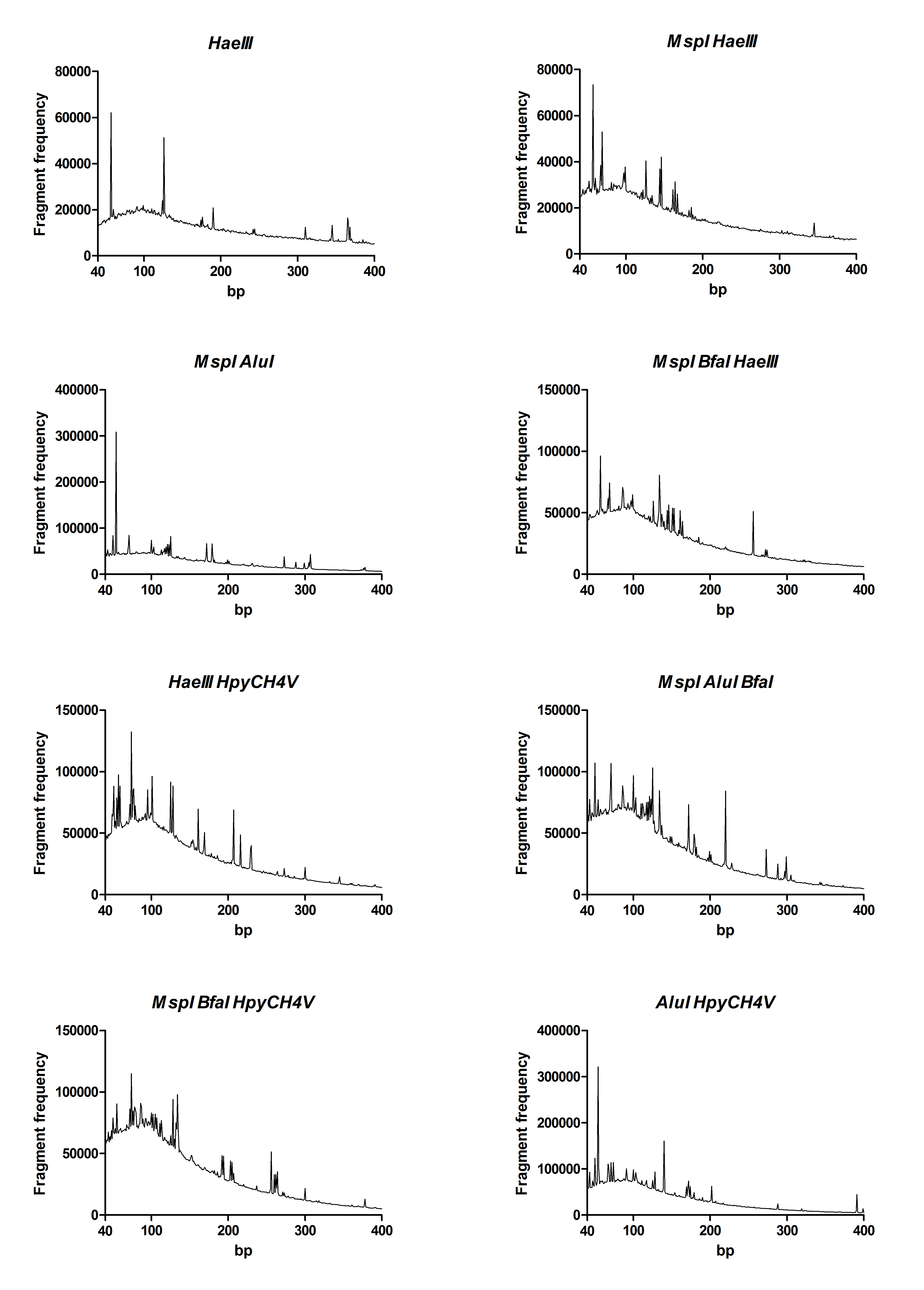

Supplement: Supplementary file 3 — Additional file 3: Figure S2: Fragment distribution of selected enzymes in Rattus novergicus. (TIFF 2 MB) [file 13104_2014_3055_MOESM3_ESM.tiff]

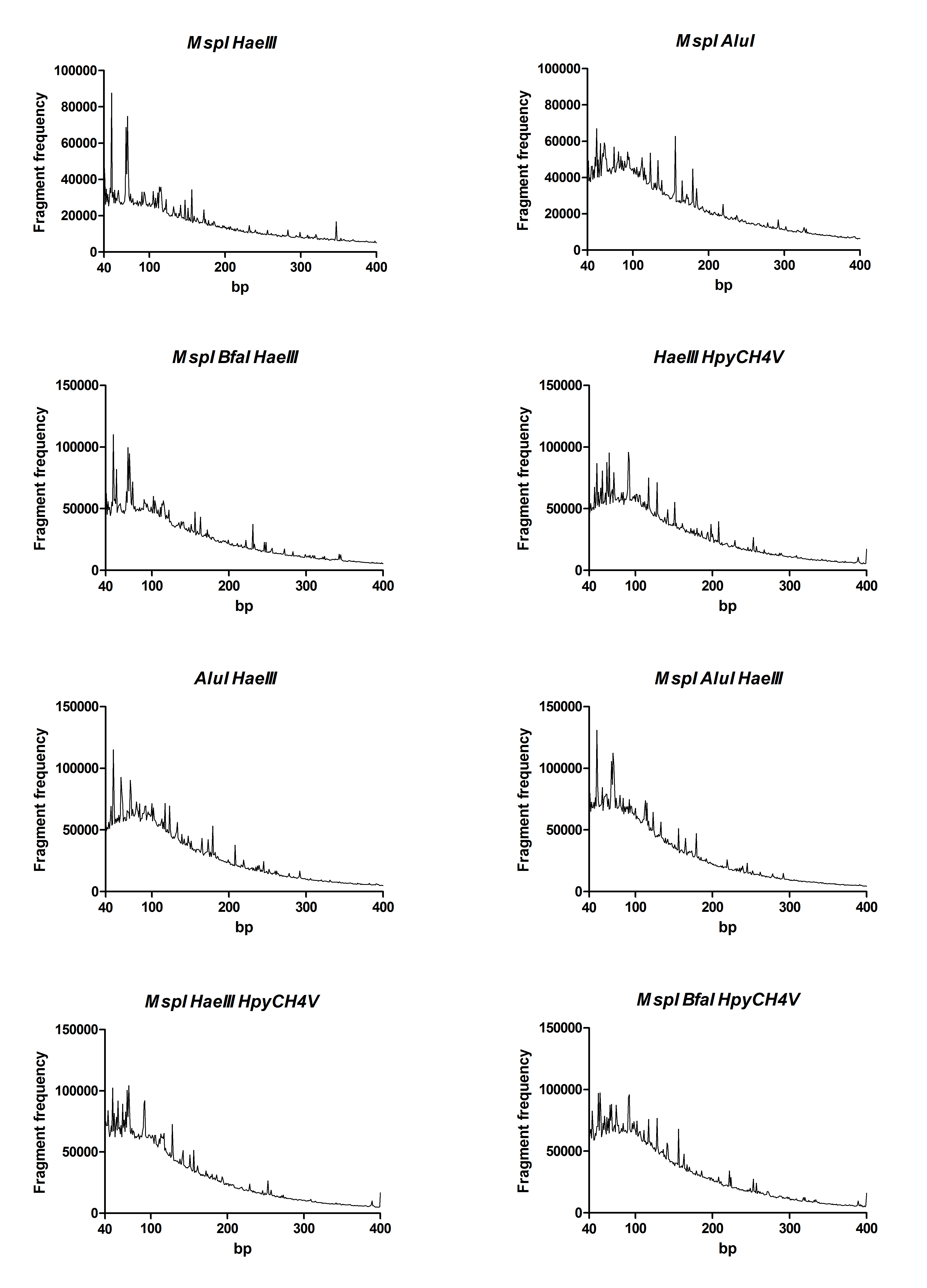

Supplement: Supplementary file 4 — Additional file 4: Figure S3: Fragment distribution of selected enzymes in Mus musculus. (TIFF 2 MB) [file 13104_2014_3055_MOESM4_ESM.tiff]
